# Actinomycosis of the Udder

**Published:** 1898-02

**Authors:** 


Actinomycosis of the Udder.—A heifer, two and one-half years of age, presented abundant salivation and a swelling of the parotid gland, which was relieved by the local application of tincture of iodine.

Two months afterward a mammitis of one of the quarters of the udder was discovered. The general condition gradually became worse and she rapidly became cachectic. The animal was killed.

At the necropsy numerous actinomycotic centres were found in the parotid gland and in the inflamed quarter of the udder. The author thinks that a cure could have been effected with iodide of potash, if a correct diagnosis had been made.

Erhardt never observed an instance of the transmission of actinomycosis to neighboring animals.—*Ann. de Méd. Vét.*, November, 1896.

